# Polypharmacy Leading to Priapism in HIV Patient with Schizoaffective Disorder: A CYP450 Cascade

**DOI:** 10.1155/2019/4562065

**Published:** 2019-01-03

**Authors:** Vinod Sharma, Aditi Sharma

**Affiliations:** Southern Illinois University Child Psychiatry, P.O. Box 19674, Springfield, IL 62794, USA

## Abstract

With increasing trend of polypharmacy, there are higher chances of drug-drug interactions leading to adverse effects, especially in psychiatric patients with co-morbid chronic medical problems. This case demonstrates a schizoaffective 30-year-old male on highly active antiretroviral therapy (HAART) who reported an incident of priapism potentially caused by an interaction between previously prescribed atypical antipsychotic, trazodone, norepinephrine dopamine reuptake inhibitor (NDRI), HAART, and newly added selective serotonin reuptake inhibitor (SSRI). This case emphasizes obtaining careful medication history, understanding cytochrome P450 (CYP450) enzyme and its subtypes, and discouraging polypharmacy. Clinicians must educate their patients about different sexual side effects as these can be socially stigmatizing and can further deteriorate mental health symptoms.

## 1. Introduction

Priapism is defined as persistent erection of penis or clitoris that is not associated with sexual stimulation or desire. The duration of erection varies among studies, but most defined it for more than four hours. The most common cause of priapism is idiopathic, and other known causes include medications like antihypertensive drugs, selective serotonin reuptake inhibitors (SSRIs), phenothiazine, trazodone, and haloperidol; medical disorders like sickle cell disease (SCD); and illicit drugs like cocaine. The most common cause of priapism is medications in adults and SCD in children [1]. This is a case report of 30-year-old HIV-positive Caucasian male with history of schizoaffective disorder that developed painful penile erection after a single dose of sertraline 50 mg. In this case, we will discuss about different psychotropic medications and their possible interactions among themselves and with highly active antiretroviral therapy (HAART) medications that could potentially lead to priapism.

## 2. Case Description

A 30-year-old Caucasian male with history of schizoaffective disorder and HIV reported an episode of painful penile erection after he took a single dose of sertraline 50 mg. As reported by patient, he did not seek any medical attention and it subsided on its own within 5-6 hours. He was also taking trazodone 50 mg, bupropion 450 mg, and aripiprazole 10 mg for the past few years. He denied any previous episode of painful or painless penile erection. During the previous visit, sertraline 50 mg was added to target his depressive symptoms. After this episode of priapism, patient stopped taking sertraline and it did not happen again. His HIV medications include dolutegravir (Tivicay) 50 mg daily and emtricitabine/tenofovir disoproxil (Truvada) 200/300mg. He was very distressed and embarrassed with this episode of priapism. He denies any substance abuse or other medical problems. He denies suicidal or homicidal thoughts. His labs were checked and were unremarkable. His CD4 count was 514. He did not have any risk factors like sickle cell disease, oncological malignancy, blood dyscrasias, penile trauma, pelvic injury, or prior episode of priapism.

## 3. Discussion

Drug induced priapism accounts for almost 30% of cases, the second most common cause after idiopathic cases. Priapism is an uncommon side effect of certain psychotropic drugs, particularly antipsychotics and trazodone. This can occur in patients shortly after they have been started on the antipsychotic medications, or in those who have been on them for a long time without dosage change. Sometimes, priapism occurred with the addition of another antipsychotic, lithium, or serotonin specific reuptake inhibitor [2]. The most common mechanism for drug-induced priapism is via alpha-adrenergic receptor antagonism especially alpha-1. SSRIs as a group have little affinity for alpha-1-adrenergic receptors and rarely cause priapism; however SSRI sertraline has higher affinity for alpha-1 adrenergic receptors than other SSRIs [3]. In this case, patient was already on trazodone which has alpha-1 antagonistic properties that is hypothesized to cause priapism. Also, there are reports of priapism with antipsychotics including aripiprazole, although it has the weakest affinity to alpha-1 adrenergic receptors. Therefore, addition of sertraline led to increased levels of trazodone and aripiprazole because of shared CYP450 and increase in alpha-1 antagonism which caused priapism. However, priapism is not a dose-dependent side effect [4].

Another possible explanation for drug-induced priapism is via stimulation of serotonin 5-HT 1B and 5-HT 1C/1D receptors or inhibition of 5-HT1A and 5-HT2 receptors. Since serotonin may have both stimulatory and inhibiting effects on penile erection, the outcome will depend upon which type of 5-HT receptor is involved. Sertraline induced priapism most likely results from inhibition of CNS reuptake of serotonin [4].

Drugs metabolized by CYP450 system has potential for pharmacokinetic drug interaction when they are administered with non-nucleoside reverse transcriptase inhibitors (NNRTIs) and protease inhibitors (PI) [Table 1]. Among HAART medications, PI like lopinavir and ritonavir are substrate of CYP3A4, and ritonavir is strong inhibitor of CYP2D6 and 3A4. The data is limited for dolutegravir which is an integrase inhibitor and tenofovir/emtricitabine which are nucleoside analog reverse transcriptase inhibitor (NRTIs) as they are not metabolized by CYP450 complex [5]. The addition of antidepressants to an HIV infected patient is of particular concern as both are metabolized by CYP450. The major CYP450 isoenzymes affected include CYP3A4, CYP2D6, and CYP2C9/2C19 [6]. Many SSRIs including fluoxetine, citalopram, paroxetine, and sertraline are metabolized via the CYP450 system. Also, antiretroviral agents that inhibit CYP450 metabolism, such as ritonavir, may inhibit SSRIs metabolism and increase concentrations. Sertraline is a weak inhibitor of CYP3A4 and CYP2D6 enzymes at low doses and potentially can raise the plasma level of the medications that are metabolized by CYP3A4 and CYP2D6, trazodone (by 2D6), and aripiprazole (by 2D6 and 3A4) in this case [4]. Aripiprazole is a substrate of both CYP3A4 and CYP2D6; therefore, combining with inhibitors of either enzyme may lead to increased risk for adverse effects due to aripiprazole accumulation [6]. Trazodone is metabolized by CYP3A4 and CYP2D6, leaving the potential for increased levels of trazodone when it is combined with enzyme-inhibiting protease inhibitors or NNRTIs, which can cause priapism in high doses [4, 7]. Bupropion which is commonly used in depression is inhibitor of CYP2B6 that can also potentially increase the level of trazodone and aripiprazole, further leading to priapism. In vitro, data suggests that antiretroviral agents including ritonavir, efavirenz, and nelfinavir have substantial CYP2B6 inhibition possibly resulting in increased bupropion concentrations [5].

## 4. Conclusion

It is extremely difficult to predict priapism in any case as it can occur during every stage of treatment due to use of any psychotropic drug in addition to antipsychotic. It is not possible to predict which patient will develop priapism; therefore, clinicians should be vigilant about the sexual side effects as these can be embarrassing and stigmatizing in nature. Health professionals should be careful in patients who are already on medications that are known to cause priapism [4]. Special care should be given to drug interactions when an enzyme inducing agent is discontinued. Toxicity may then ensue due to persistence of the high dose of the drug that was formerly needed to offset the inducing effect [5]. They should also educate their patients about seeking medical help if any of these sexual side effects appear. Just like alpha-2A adrenergic receptor gene polymorphism has been associated with sialorrhea on clozapine. Could alpha-1 adrenergic receptor gene share the same polymorphism in psychotic patients to increase the chances of priapism is a topic for future research. Further investigation is also required regarding potential interactions between HAART, SSRIs, and anti-psychotics to elucidate potential mechanisms of adverse effects.

## Figures and Tables

**Table 1:** List of drugs metabolized by cytochrome P450 enzymes.

**Drug**	**Substrate**	**Inducer**	**Inhibitor**
Sertraline	2D6,2C9/19	-	2D6(weak)

Bupropion	2B6	-	2D6

Trazodone	3A4,2D6	-	-

Aripiprazole	3A4,2D6	-	-

**NNRTIs**			
Nevirapine	3A4, 2B6	3A4	3A4,2D6,2C9/19
Efavirenz	3A4, 2B6	3A4	3A4,2D6,2C9/19
Delaverdine	3A4, 2B6	3A4	3A4

**Protease inhibitors**			
Ritonavir	3A4, 2D6	2C9/19, 1A2	2D6, 3A4 (strongest)
Indinavir	3A4, 2D6	-	3A4
Nelfinavir	3A4, 2D6	-	3A4
Squanavir	3A4, 2D6	-	3A4 (weakest)
